# MWF of the corpus callosum is a robust measure of remyelination: Results from the ReBUILD trial

**DOI:** 10.1073/pnas.2217635120

**Published:** 2023-05-08

**Authors:** Eduardo Caverzasi, Nico Papinutto, Christian Cordano, Gina Kirkish, Tristan J. Gundel, Alyssa Zhu, Amit Vijay Akula, W. John Boscardin, Heiko Neeb, Roland G. Henry, Jonah R. Chan, Ari J. Green

**Affiliations:** ^a^Department of Neurology, UCSF Weill Institute for Neurosciences, University of California, San Francisco, CA 94143; ^b^Department of Brain and Behavioral Sciences, University of Pavia, 27100 Pavia, Italy; ^c^Department of Medicine, University of California, San Francisco, CA 94143; ^d^Department of Epidemiology & Biostatistics, University of California, San Francisco, CA 94143; ^e^Multimodal Imaging Physics Group, Department of Mathematics and Technology, Koblenz University of Applied Sciences, 53424 Koblenz, Germany; ^f^Institute for Medical Engineering and Information Processing, University of Koblenz and Landau, 56070 Koblenz, Germany; ^g^Department of Ophthalmology, University of California, San Francisco, CA 94143

**Keywords:** **r**emyelination, myelin water fraction, multiple sclerosis, MRI

## Abstract

Myelin repair is an unrealized therapeutic goal in the treatment of multiple sclerosis (MS). Uncertainty remains about the optimal techniques for assessing therapeutic efficacy and imaging biomarkers are required to measure and corroborate myelin restoration. We analyzed myelin water fraction imaging from ReBUILD, a double-blind, randomized placebo-controlled (delayed treatment) remyelination trial, that showed a significant reduction in VEP latency in patients with MS. We focused on brain regions rich in myelin. Fifty MS subjects in two arms underwent 3T MRI at baseline and months 3 and 5. Half of the cohort was randomly assigned to receive treatment from baseline through 3 mo, whereas the other half received treatment from 3 mo to 5 mo post-baseline. We computed myelin water fraction changes occurring in normal-appearing white matter of corpus callosum, optic radiations, and corticospinal tracts. An increase in myelin water fraction was documented in the normal-appearing white matter of the corpus callosum, in correspondence with the administration of the remyelinating treatment clemastine. This study provides direct, biologically validated imaging-based evidence of medically induced myelin repair. Moreover, our work strongly suggests that significant myelin repair occurs outside of lesions. We therefore propose myelin water fraction within the normal-appearing white matter of the corpus callosum as a biomarker for clinical trials looking at remyelination.

## Significance

Putative imaging biomarkers for myelin in MS have been correlated with pathological evidence of myelin loss in the context of a complex pathological environment. However, they have not been shown to improve dynamically with myelin repair because of limited therapies available that can induce that repair. Using MRI analysis from the double-blind, placebo-controlled remyelination ReBUILD trial we provide the first direct, biologically validated imaging-based evidence of medically induced myelin repair. MRI-derived myelin water fraction (MWF) values increase in normal-appearing white matter of the corpus-callosum with the administration of a tool remyelinating compound (clemastine). Furthermore, significant myelin repair occurs outside of lesions—focusing attention for repair beyond the lesion and setting corpus-callosum MWF as a standard for clinical programs investigating remyelinating therapies.

Restoration of the myelin sheath is an unrealized therapeutic goal in the treatment of multiple sclerosis (MS) that promises functional recovery and prevention of long-term disability (1–3). Demyelination of axons—especially if persistent—is believed to be injurious to neurons and serves as a major contributor to the irreversible cell loss that underlies permanent disability (4).

Available MS treatments block access of immune cells to their target tissue or otherwise attenuate the inflammatory response without directly addressing or fully preventing axonal degeneration and disability. An encouraging therapeutic approach for remyelination is to enhance differentiation of the endogenous oligodendrocyte precursor cells (OPCs) into mature myelinating oligodendrocytes and thereby stimulate remyelination of demyelinated axons before the neuronal substructure is permanently lost (5).

There are currently no validated imaging methods for structurally demonstrating myelin restoration (6). Despite success in identifying promising therapeutic candidates capable of inducing OPC differentiation and consequent remyelination, much uncertainty remains about the optimal techniques for assessing treatment efficacy. Given the clinical imperative to advance remyelinating treatments in the clinic, biomarkers for measuring myelin repair are needed. This necessitates unequivocal confirmation of a potential biomarker’s utility for measuring myelin status. MRI sequences that have been purported to measure myelin, such as magnetization transfer ratio (MTR) (7, 8), MTsat (9), and myelin water fraction (MWF) (10–12), actually measure pools of large macromolecules or water within myelin lamellae, as a proxy. Conventional diffusion methods have also been extensively used in the field, but they lack specificity to myelin (13). Advanced diffusion models [e.g., NODDI (14)] as well as combination of imaging modalities used to estimate in vivo the g-ratio (15) have recently been described as possible methods to study myelin, but further validation studies are needed (13). Some of these techniques have been proposed and even asserted to serve as sensitive biomarkers of myelin status (6). The capacity of these methods to measure myelin has been largely based on their correspondence with myelin loss (11, 16, 17) or interpretation of pathologically partially demyelinated lesions as remyelinated rather than only partially demyelinated—a tenet that has recently been brought into question (18). In addition, variations in signal intensity seen with these methods across different regions of the brain (19, 20) has additionally provided support to the concept that they measure myelin. However, these methods have not been demonstrated to be responsive to treatment with a remyelinating agent as evidence of myelin recovery.

This study is based on the evaluation of the myelin-dedicated MRI data from the double-blind, placebo-controlled trial (ReBUILD) which reached its primary endpoint to document remyelination (21) and took place once all ReBUILD data analysis was completed. Within the ReBUILD trial predefined secondary imaging endpoints, also including new and enlarging lesions on T_2_-weighted imaging and volume of gadolinium-enhancing lesions on T_1_-weighted imaging, whole brain MWF showed no evidence of improvement while on the study drug (21). The specific MWF method based on T_2_^*^ relaxation used in the trial (22) was chosen among the several potential MRI methods to assess myelin based on two main factors: the higher specificity of the method to myelin (13, 23, 24) and its clinical feasibility [availability of the MRI protocol for our scanner and acquisition time (<10 min)].

Considering the amount of noise related with this measure and the difficulties due to the necessity to analyze MWF in poorly myelinated areas, however, we decided to analyze the same measure in highly myelinated areas (25), in particular, focusing on the corpus callosum (CC) that animal models studies identified as promising candidate for assessing remyelination (26). The aim was to help define the potential for MRI to capture remyelination in MS, determine the optimal sequences and location for measuring myelin recovery and to help guide trial design for future reparative and remyelinating trials.

## Results

**Fig. 1.**
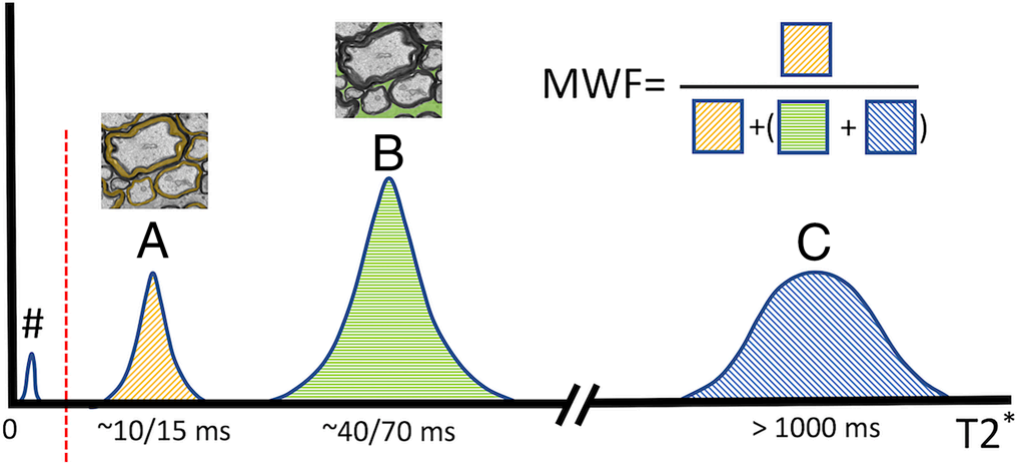
Illustrative T_2_^*^ spectrum of MRI proton signal in a brain voxel: “#” represents myelin protons that don’t contribute to MRI signal due to very short T_2_^*^; “A” represents the water protons in the myelin layers restricted by the myelin sheaths; “B” represents the protons of intra- and extracellular water having intermediate T_2_^*^ values; “C” represents cerebrospinal fluid and unrestricted water pools characterized by much longer T_2_^*^ values. The approach used in this study computes the myelin water fraction (MWF) deriving the area of the peak “A” without differentiating the “B” and “C” pools whose combined area is estimated.

We analyzed the myelin content dedicated imaging of the ReBUILD clinical trial participants (50 patients) and computed MWF (Fig. 1) changes occurring in the NAWM and lesional WM of three highly myelinated selected regions [CC, optic radiations (OR), and corticospinal tracts (CST)]. Study randomization was previously described in detail (21); half of the cohort was randomly assigned to group 1 and received treatment from baseline through 3 mo, whereas the other half (group 2) received treatment from 3 mo to 5 mo post-baseline.

**Fig. 2.**
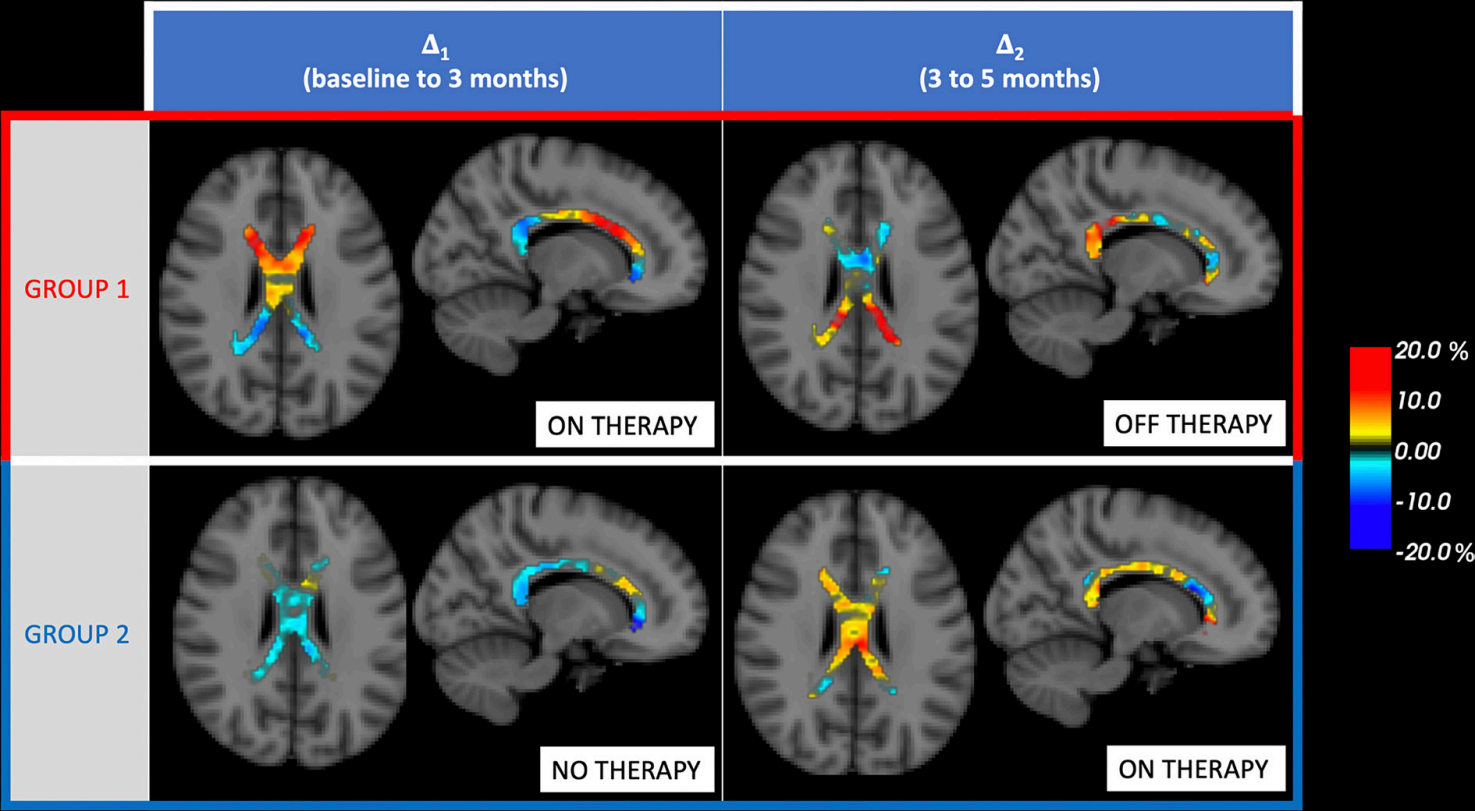
Treatment-related MWF changes within the CC. Figure shows voxel-based monthly percentage changes “Δ” in CC: baseline to 3 mo “Δ_1_“; 3 to 5 mo “Δ_2_” for groups 1 and 2. Group median values were computed voxel-wise.

Based on the mixed effects linear modeling of the MWF values in the CC, the two groups values were comparable at baseline (group 1: mean 0.087, 95% CI [0.080, 0.095]; group 2: mean 0.088 [0.081, 0.096], *P* = 0.9 for group difference). At 3 mo, group 1 mean MWF increased to 0.092 [0.084, 0.100], while group 2 mean WMF decreased to 0.082 [0.074, 0.090], *P* = 0.012 for difference in change from baseline. Finally, at 5 mo, group 1 mean MWF continued to increase to 0.094 [0.087, 0.102], while group 2 mean MWF increased by a similar amount up to 0.086 [0.078, 0.094], *P* = 0.032 for difference in change from baseline (Fig. 2 and Table 1).

**Table 1.** Group 1 and group 2 MWF values within each volume of interest and lesions are reported as mean, inferior (lcl95), and superior (ucl95) 95% CI at baseline, 3 mo, and 5 mo

Table 1		Group 1			Group 2			Diff
		mean	lcl95	ucl95	mean	lcl95	ucl95	*P*
MWF in corpus callosum NAWM	baseline	0.087	0.080	0.095	0.088	0.081	0.096	0.872
	3 mo	0.092	0.084	0.100	0.082	0.074	0.090	0.012
	5 mo	0.094	0.087	0.102	0.086	0.078	0.094	0.032
MWF in optic radiation NAWM	baseline	0.078	0.072	0.083	0.077	0.071	0.083	0.847
	3 mo	0.080	0.074	0.083	0.075	0.069	0.081	0.340
	5 mo	0.080	0.074	0.085	0.075	0.070	0.081	0.372
MWF in corticospinal tract NAWM	baseline	0.092	0.086	0.099	0.090	0.083	0.097	0.757
	3 mo	0.090	0.083	0.097	0.090	0.083	0.097	0.762
	5 mo	0.089	0.082	0.096	0.091	0.084	0.098	0.573
MWF in WM lesions	baseline	0.065	0.056	0.073	0.072	0.064	0.081	0.194
	3 mo	0.071	0.062	0.079	0.069	0.061	0.078	0.213
	5 mo	0.073	0.065	0.082	0.065	0.057	0.074	0.035

No statistically significant changes were observed in the OR and CST VOIs (Table 1).

Regarding the analysis of lesion MWF, at baseline versus 3 mo, there was no significant difference in MWF change from baseline between group 1 and group 2 (Table 1). At 5 mo, there was a modest but statistically significant difference in MWF change from baseline between group 1 and group 2 (*P* = 0.035).

Four patients’ measurements (2 from the third month timepoint, 2 from the fifth month timepoint, all belonging to group 1) were excluded from the dataset for poor quality due to excessive patient motion during acquisition. For this reason, the analysis was based on 94 out of 100 calculated interval changes.

The reliability assessment of the MWF processing based on seven healthy subjects indicated no statistical differences between the three MWF datasets, using ANOVA. The average coefficient of variation was 5.36% in the CC, 3.44% in the OR, and 8.49% in the CST.

The subset analysis performed in all VOIs with a reliable H_2_0 map showed the benefit of clemastine on MWF in the CC and confirmed that the absolute H_2_0 content was stable throughout the trial.

Assuming an improvement in mean MWF of the CC from baseline of 0.01 (similar to what was observed), the power analysis suggests that 58 patients per arm would need to be enrolled for a clinical trial of a similarly efficacious drug with a parallel arms study design assessing MWF in the CC (with a standard 80% power and a two-sided alpha = 0.05). In contrast, the observed difference in mean from baseline for OR and CST was lower (Table 1) than 0.01. Therefore, for assessing change in MWF in the OR, considering a 0.005 difference (similar to what was observed), at least 94 subjects per arm would need to be enrolled, while given the lower detected difference for CST at least 200 patients per arm would be needed for studying this tract. All these analyses assume a similar magnitude of effect for a theoretical study drug compared to clemastine as well as power = 80% and a two-sided alpha of 0.05. Adjustments in sample size would need to be made based on drug effect size and to adjust power as desired. Additional increases in sample size may also be required to account for study dropout in any future theoretical study given the exceptionally low dropout seen in ReBUILD.

Furthermore, we found a negative correlation (*P* = 0.027) between OR MWF change and VEP change. We also found a negative correlation between CC MWF and VEP change that does not reach statistical significance (*P* = 0.11).

## Discussion

We present the first positive identification of an MRI correlate of therapy-induced remyelination in a clinical trial (ReBUILD) (21) with a technique that can be employed in a clinically feasible time. Patients in both groups exhibited an increase in MWF values within the normal-appearing white matter (NAWM) of the corpus callosum while on the active compound (relative increase of 4.5% and 4.4% in group 1 and group 2, respectively). The increase in MWF was also sustained into the second epoch of group 1 (relative change of 3.1%) (Fig. 2). There was a decrease in MWF in group 2 (relative decrease in MWF of 6.2%) while not on treatment. The CC and OR MWF change both showed a negative correlation with VEP change (meaning shortened latency with higher MWF in those regions), significant within OR VOI. This result strengthens the interpretation that MWF allows for quantification of therapeutically induced remyelination and might suggest that remyelination in the posterior visual pathway contributes to the latency improvement seen.

We were able to successfully measure an improvement of the myelin content metrics in the CC NAWM of subjects enrolled in the ReBUILD trial (21). The corpus callosum is widely known as a sensitive area to test demyelination in toxic animal models (26), and therefore serves as a strong candidate for assessing remyelination in human subjects. We also decided to analyze two less promising but important and well-myelinated pathways (25): the optic radiation and the corticospinal tract. MWF in the optic radiation showed a similar trend to CC (despite being not statistically significant), whereas no treatment-correlated changes were observed within the corticospinal tract region of interest. This may indicate a regional tract-specific effect of the treatment or may be related to technical issues, specifically a misregistration of the optic radiation and the corticospinal tract, compared to a highly coherent white matter structure such as the corpus callosum.

Since its earliest description, the primary focus in MS has been in understanding focal lesions (27, 28) and preventing them from occurring—in fact all existing approved disease-modifying therapies for MS have been approved based on their capacity to prevent the formation of new lesions and reduction in the rate of their associated clinical relapses. Moreover, myelin repair research has remained focused on studying myelin restoration in the context of the lesion on the presumption that it is both more readily measurable and functionally salient. Nevertheless, myelin repair was captured by our MRI analysis in normal-appearing WM, whereas unequivocal treatment-related changes were not present within lesions. This difference is likely due to the presence of partially unmyelinated axons within the NAWM that can still take advantage of a remyelinating treatment. Plaques, on the other hand, are characterized by denuded surviving axons embedded in variable amounts of astroglial scar tissue, macrophages, and infiltrating lymphocytes. The low number of demyelinated axons available to remyelination outside the matrix of scarring (fibrous) astrocytes as well as the loss of axons and unknown factors related to the persistent inflammation likely explain why lesions offer a reduced substrate for remyelination compared to NAWM. Furthermore, MS lesions are intrinsically heterogeneous due to diverse pathological processes underlying different focal lesion types (29) as well as due to lesion evolution that occurs with disease progression. Recent work has highlighted the significant pathological injury that occurs in MS in the so-called normal-appearing white matter (29). This diffuse inflammatory reaction is similar to what is seen within lesions including perivascular cuffs of mononuclear cells, diffuse infiltration of the tissue by T-lymphocytes and profound microglia activation (30).

Importantly at a histopathological level, MS NAWM demonstrates global reduction in the intensity of myelin staining but also focal axonal swellings and axonal end bulbs. This axonal damage is more prominent around demyelinated plaques and within defined tracts emerging from the plaques. Neuronal loss in NAWM increases in progressive patients, highlighting a dynamic picture characterized by unmyelinated and damaged axons prone to cell death, opposed to the stability of the axonal loss within the plaque.

As myelin is laid down and compacted water in the form of cytoplasm and extracellular water is expelled leaving only a very thin layer of cytoplasmic water (approximately 2 nm) and extracellular water (approximately 4 nm between each wrap) remaining (23, 31). These thin layers of water within myelin lamellae are postulated to be the primary source of the MWF signal. Myelin water represents only about 10% of the total signal within a voxel; therefore, noise as small as few percent of the total signal affects MWF quantification. Importantly, MWF quantification can be affected because of a decrease in the other water signal, potentially due to a reduction of global inflammation caused by treatment. To exclude this possibility, our group has performed extensive work showing that clemastine does not have an impact on inflammation in animal models of inflammatory demyelination. Infiltration of T cells and macrophages and activation of microglia in demyelinated lesions at the late stage of EAE are in fact not modified by clemastine (5). Furthermore, EAE induced in a transgenic animal model characterized by deletion of the muscarinic acetylcholine receptor 1 (target of clemastine) on NG2 positive cells, showed the same clinical score of EAE mice treated with clemastine, excluding an off-target effect of the drug (5). We additionally unequivocally demonstrated that the effect on the VEP latency is given by an effect on myelin status, dissecting out the role of inflammation both using a chemical demyelinating animal model and a transgenic approach (32). Furthermore, our finding that H_2_O maps were stable across the study confirms that the effect of clemastine on MWF is not due to a decrease in absolute H_2_O, mediated via inflammation or some other independent mechanism.

MWF of the NAWM was recently shown to be improved with the use of ocreluzimab in a substudy of the OPERA II trial (33) using a multicomponent-driven equilibrium single-pulse observation of T_1_/T_2_ (mcDESPOT) (34). This result may reflect either that the use of certain highly potent DMTs enable a more permissive environment for remyelination or—akin to the point highlighted above—that when assessing MWF using an immunosuppressive (and presumably anti-inflammatory) agent the impact on total water must be considered. The MWF protocol implemented in the ReBUILD study was based on a gradient-echo sequence, since it allows a whole brain acquisition feasible in a clinical setting (acquisition time <10 min). Gradient-echo acquisitions are susceptible to subject motion, magnetic field inhomogeneity and inflow artifact (35). To test the precision of our myelin measurement, we additionally performed a dedicated reliability assessment of the MWF in healthy subjects; this assessment consisted in three repeated acquisitions and demonstrated reasonable precision of the MWF measurement. The capacity to detect the response to remyelinating therapy in this instance suggests the robustness of the technique rather than strictly a limitation. Future endeavors to further optimize MWF imaging of the corpus callosum should be capable of yielding improvements in image acquisition of this focused area.

In conclusion, future reparative and remyelinating trials focused on restoring function should identify myelin-content–related changes on MRI within the NAWM and especially within highly myelinated coherently organized structures like the corpus callosum as imaging endpoints. Furthermore, myelin repair appears to be more robust in the NAWM than in the lesion which should focus our attention on understanding myelin injury in the NAWM and its impact on disease.

## Materials and Methods

We analyzed the MRI data from all fifty relapsing-remitting patients enrolled in the ReBUILD trial [average age of 40.1 y ± 10 SD, 64% female, median (range) EDSS 2 (0 to 5.5), and average disease duration of 5.1 y ± 5 SD]. Demographic and clinical characteristics were previously reported in detail (21). ReBUILD trial subjects underwent MRI at three time points (at baseline, months 3 and 5) on a 3T Siemens Skyra scanner equipped with a 20-channel head/neck coil. Details of the MRI protocol were previously reported (21). It included standard sagittal 3D T_1_-weigthed MPRAGE and T_2_-weighted FLAIR acquisitions (both 1-mm^3^ voxel), and a MWF protocol. In detail, the MWF protocol consisted in a volume acquired using an axial 2D multigradient-echo FLASH sequence (in-plane resolution = 1.25 × 1.25 mm^2^, field of view (FOV) = 240 × 240 mm^2^, 50 slices of 2.5 mm thickness) with a flip angle of 40°, repetition time (TR) = 2,260 ms and total acquisition time of 4:15 min. For each acquisition, 10 echoes spaced 4 ms apart were collected [first echo time (TE) = 4.70 ms, last TE = 40.70 ms]. To compute T_1_ relaxation times and correct for signal saturation effects, a second axial 2D FLASH volume was acquired (TR = 700 ms, two echoes with TE = 4.70 and 8.70 ms, flip angle 70°) (22). A series of low-resolution GRE-EPI were also acquired to correct for B0 and B1 radio frequency inhomogeneity. The MWF protocol had a total acquisition time of about 9 min.

**Fig. 3.**
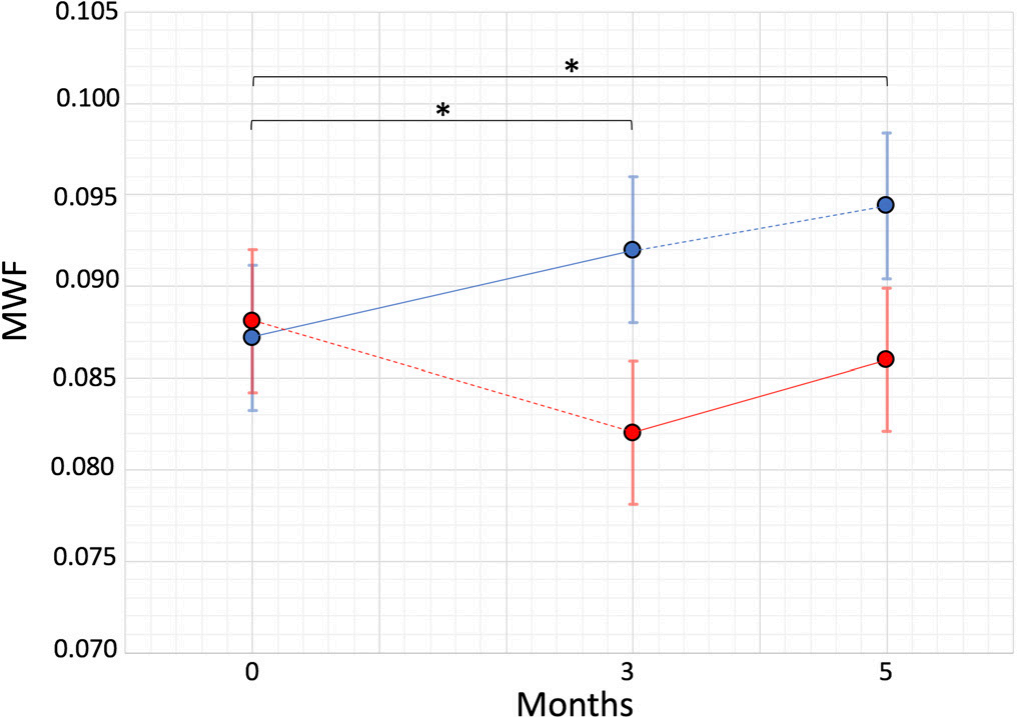
Myelin water fraction (MWF) of the corpus callosum at baseline, 3-mo, and 5-mo visits. Blue = Group 1 (immediate treatment), Red = Group 2 (delayed treatment), and on-treatment epochs are denoted by a solid line. A significant difference in change from baseline between groups (marked by “*”) was seen at 3 mo (*P* = 0.012) and 5 mo (*P* = 0.032).

At Each of the Three Time Points, a MWF Map Was Computed for ReBUILD. Study Subjects. Similarly, a MWF was generated for the three consecutive acquisitions on seven healthy subjects (see below). For MWF computation, we applied the method by Neeb and colleagues (Fig. 3) (22). MWF maps and FLAIR images were coregistered to the corresponding T_1_ space, using linear and nonlinear transformations of FSL software library (FLIRT/FNIRT) (36, 37). An additional linear and nonlinear coregistration was performed between the T_1_ MPRAGE images and the MNI152 standard-space T_1_-weighted average structural template image, in order to transform/transfer the MWF maps and FLAIR images to the same MNI152 standard-space. The MNI space, white matter, and probabilistic atlas (ICBM-DTI-81) (36) were used to obtaine volume of interest (VOIs) of the CC, OR, and CST. A conservative threshold was applied to the atlas VOIs to focus analysis on the core of the pathways, avoiding partial volume averaging with surrounding structures. All the processed images were visually inspected for quality confirmation. An expert neuroradiologist (EC) blinded to any other study data segmented white matter lesions from FLAIR at baseline. Baseline white matter lesion segmentation was used as a mask for assessing changes throughout the study. No change in lesion burden (both number and volume) was observed within the 5-mo interval in any subject. The segmented lesions were subtracted from the total white matter to obtain the NAWM. Finally, the median value of MWF was computed for each NAWM VOI as well as for lesions. All the MRI processing was performed blind to any subject information. Once these values were obtained, the differences between time-point values were computed (delta change, D) and the data was then analyzed based on group assignment.

The study was approved by the UCSF Institutional Review Board and all participants provided informed consent. The trial was registered at ClinicalTrials.gov (number NCT02040298) before initiation of patient enrolment. All research was performed in accordance with relevant guidelines/regulations.

### Reliability and Test–Retest Variability Analysis.

In order to assess the reliability and test–retest variability of the MWF data, we additionally acquired MRI data on seven healthy subjects (4F, average age± SD of 25 y ± 2). The dedicated reliability measurement assessed the test–retest variability of the MWF data within the corpus callosum. It was based on the acquisition of three consecutive, complete, 2D FLASH acquisitions for each of the seven healthy subjects. This included interval subject repositioning where the MRI technician took the subjects off and repositioned them on the scanner bed.

The MRI acquisition and processing method we used in this study provides both absolute H_2_O content maps as well as MWF maps, independently computed. In order to confirm that the MWF change was not indirectly due to absolute water content changes, we undertook a further imaging analysis evaluating both the MWF and the absolute H_2_0 in all subjects and in all VOIs where we had a reliable H_2_0 map,

## Statistical Analysis.

Group 1 received treatment from baseline through 3 mo and group 2 from 3 mo to 5 mo post-baseline. Given the structural change induced by the treatment, a delayed treatment analysis was undertaken to evaluate the effect of treatment on the MRI outcomes. Mixed-effects linear models were used to compare the changes from baseline at each time point between the two groups in the NAWM and lesions of the CC, OR, and CST. More specifically, the model specified the categorical time point, the treatment indicator (1 for months 3 and 5 in group 1 and 1 for month 5 in group 2), and the interaction of these as fixed effects and included a random intercept for each subject to account for intrasubject correlation of the longitudinal measurements.

To examine the supplementary dedicated reliability dataset (based on the MWF values within the VOIs of the seven healthy subjects), we calculated the median MWF from each VOI for each subject and time point. We performed an ANOVA for the three time points and computed coefficient of variation for each subject. We report the average coefficient of variation across subjects for each VOI.

The correlation between CC and OR MWF and VEP change was assessed using a hierarchical linear model with random intercepts and slopes, accounting for within-subjects and within-eye correlations.

To consider planning for future trials we performed a power analysis for the different tracts we used the SD within groups at baseline and expected difference in mean change from baseline, ranging from 0.005 to 0.01 (similar to the differences in mean change from baseline observed in the trial in the different VOIs).

## Data Availability

All data and materials (including data tables, analysis code and scripts) used for this paper have been deposited in the public repository Dryad for any researcher to reproduce or extend the analysis, except for information that may compromise data anonymization or subject privacy (38).
